# Functional Activity of Monocytes and Macrophages in HTLV-1 Infected Subjects

**DOI:** 10.1371/journal.pntd.0003399

**Published:** 2014-12-18

**Authors:** Camila F. Amorim, Anselmo S. Souza, Angela G. Diniz, Natália B. Carvalho, Silvane B. Santos, Edgar M. Carvalho

**Affiliations:** 1 Serviço de Imunologia, Complexo Hospitalar Universitário Professor Edgard Santos, Universidade Federal da Bahia, Salvador, Bahia, Brazil; 2 Departamento de Ciências Biológicas, Universidade Estatual de Feira de Santana, Feira de Santana, Bahia, Brazil; 3 Instituto Nacional de Ciências e Tecnologia de Doenças Tropicais (CNPq), Salvador, Bahia, Brazil; George Mason University, United States of America

## Abstract

The Human T lymphotropic virus type-1 (HTLV-1) infects predominantly T cells, inducing proliferation and lymphocyte activation. Additionally, HTLV-1 infected subjects are more susceptible to other infections caused by other intracellular agents. Monocytes/macrophages are important cells in the defense against intracellular pathogens. Our aims were to determine the frequency of monocytes subsets, expression of co-stimulatory molecules in these cells and to evaluate microbicidal ability and cytokine and chemokine production by macrophages from HTLV-1 infected subjects. Participants were 23 HTLV-1 carriers (HC), 22 HAM/TSP patients and 22 healthy subjects (HS) not infected with HTLV-1. The frequencies of monocyte subsets and expression of co-stimulatory molecules were determined by flow cytometry. Macrophages were infected with *L. braziliensis* or stimulated with LPS. Microbicidal activity of macrophages was determined by optic microscopy. Cytokines/chemokines from macrophage supernatants were measured by ELISA. HAM/TSP patients showed an increase frequency of intermediate monocytes, but expression of co-stimulatory molecules was similar between the groups. Macrophages from HTLV-1 infected individuals were infected with *L. braziliensis* at the same ratio than macrophages from HS, and all the groups had the same ability to kill *Leishmania* parasites. However, macrophages from HTLV-1 infected subjects produced more CXCL9 and CCL5, and less IL-10 than cells from HS. While there was no correlation between IFN-γ and cytokine/chemokine production by macrophages, there was a correlation between proviral load and TNF and CXCL10. These data showed a dissociation between the inflammatory response and microbicidal ability of macrophages from HTLV-1 infected subjects. While macrophages ability to kill an intracellular pathogen did not differ among HTLV-1 infected subjects, these cells secreted high amount of chemokines even in unstimulated cultures. Moreover the increasing inflammatory activity of macrophages was similar in HAM/TSP patients and HC and it was related to HTLV-1 proviral load rather than the high IFN-γ production observed in these subjects.

## Author Summary

HTLV-1 predominantly infects T cells, inducing cell proliferation and activation. While there is a larger amount of studies regarding T cells functions in HTLV-1 infected subjects, little is known about innate immunity. We evaluated monocyte and macrophage functions in HTLV-1 infected subjects. We observed that HAM/TSP patients have an increased frequency of intermediate monocytes, but expression of co-stimulatory molecules in these cells was similar between HTLV-1 infected subjects and healthy subjects (HS). Additionally, the microbicidal ability of macrophages from HTLV-1 infected subjects to kill *Leishmania braziliensis* is preserved, and these cells showed inflammatory profile, producing more CXCL9 and CCL5, and less IL-10 than macrophages from HS. It was important to determine if the exacerbated ability of macrophages to secrete cytokine was due to IFN-γ production. While there was no correlation between IFN-γ levels by PBMCs and cytokine/chemokine production by macrophages, there was a direct correlation between proviral load and TNF and CXCL10 levels. Our data indicate that despite the high production of proinflammatory mediators, macrophages from HTLV-1 infected subjects kill an intracellular pathogen in similar levels than cells from HS and pointed out for the role of viral factors inducing the inflammatory response in these cells.

## Introduction

Human T lymphotropic virus type 1 (HTLV-1) infects about 15 to 20 million people worldwide, with endemic foci in virtually all continents [1], [2]. A large proportion of individuals remain asymptomatic until the end of life, but a subgroup of infected individuals will develop a malignant lymphoproliferative disease called adult T cell leukemia/lymphoma (ATLL) [3], [4] or a chronic neurodegenerative inflammatory disease called HTLV-1 associated myelopathy/tropical spastic paraparesis (HAM/TSP) [5]. Additionally, more than 40% of infected individuals will present clinical manifestations, such as infectious dermatitis [6], polymyositis [7], sicca syndrome [8], [9], overactive bladder and/or erectile dysfunction [10], [11], chronic periodontitis [12] and HTLV-1 associated arthropathy among other diseases [13], [14], [15]. The pathogenesis of diseases associated to HTLV-1 is related predominantly to the proviral load and the exaggerated inflammatory response in HTLV-1 infection [16], [17].

HTLV-1 infects predominantly CD4^+^ T cells, but CD8^+^ T cells [18], monocytes/macrophages [19], [20] and dendritic cells [21] can also be infected by the virus. The infection is characterized by a high spontaneous proliferation and activation of T cells, leading to high production and secretion of inflammatory mediators, such as TNF, IFN-γ, CXCL9 and CXCL10 [16], [22]. Previous immunological studies have directed attention to the role of T cells in HTLV-1 infection, and seek to correlate the dysfunctions of the adaptive immune system with the development of diseases or clinical manifestations associated with the virus. Very few studies have evaluated the role of the innate immune response in HTLV-1 infection.

It is well known that HTLV-1 infection increases susceptibility and severity to other infectious diseases [23], [24], [25]. The mechanism involved in the increased susceptibility of HTLV-1 infected subjects to other infectious agents is only partially known [23], [26]. Regarding intracellular pathogens, despite the high IFN-γ and TNF production there is an increased susceptibility to *Mycobacterium tuberculosis* [27], [28], [29] and fungal infections [30]. It is known that cells of the innate immunity response, such as neutrophils and macrophages, are important effectors cells against infectious agents. However very few studies have evaluated monocytes, macrophages or neutrophils functions in HTLV-1 infection. It is known that HTLV-1 infection results in spontaneous activation of neutrophils, as indicated by increasing in the number of positive cells in the nitroblue tetrazolium test (NBT) (indicating high *burst oxidative* activity), and by the decreasing in the number of neutrophils expressing CD62L and higher expression of CD66b [31], [32]. Regarding dendritic cells (DCs), some studies showed an increased expression of molecules involved in virus internalization process and T cells adhesion (DC-SIGN) [33], [34], and a decrease in CD14 and CD1a, molecules related with derived-monocytes DCs maturation have been described [35]. Moreover, DCs from HTLV-1 infected patients show an impaired expression of CD83, CD86 and HLA-DR after stimulation with TNF and reduced ability to stimulate T cells not infected with the virus [35]. A recent study developed in Jamaica, with a cohort of HTLV-1 infected subjects, documented a decreased frequency of plasmocytoid DCs (pDCs) and expression of HLA-DR in ATL and HAM/TSP patients compared to ACs and HC. Myeloid DCs (mDCs) also showed a lower expression of HLA-DR in HAM/TSP patients. However, the expression of CD86 in both plasmocytoid and mDCs was higher in HAM/TSP patients compared to the other groups. They also demonstrated that the programmed death ligand 1 (PD-L1) is high-expressed in DCs from HAM/TSP compared to ACs [36].

These and others dysfunctions in the myeloid cell lineage may modify the immune response of HTLV-1 infected subjects to antigens. However, studies regarding the inflammatory response and microbicidal activity of monocytes and macrophages in HTLV-1 infected subjects have not been performed. The aims of the present study were to evaluate monocytes and macrophages functions in HTLV-1 infected subjects, by comparing the frequency of monocyte subsets in HTLV-1 infected subjects and the ability of monocyte-derived macrophages from HTLV-1 infected subjects to produce cytokines and chemokines and to kill the intracellular pathogen *Leishmania braziliensis*. Moreover we evaluate if there are correlations between the frequency of monocytes subsets and cytokines/chemokines produced by macrophages with IFN-γ and proviral load in these individuals.

## Methods

### Ethical statement

All HTLV-1 subjects have been followed at the HTLV-1 clinic of the Complexo Hospitalar Universitário Professor Edgard Santos (COM-HUPES), Federal University of Bahia, Brazil. The study was approved by the Ethics Committee from the Federal University of Bahia and all patients signed a document of informed consent.

### Study design, HTLV-1 infected subjects and healthy subjects

This is a cross-sectional study with the purpose of evaluating the role of myeloid lineage cells (monocytes and macrophages) from HTLV-1 infected subjects. Participants included 45 HTLV-1 infected subjects, being 23 HTLV-1 carriers (HC), 22 patients diagnosed with HAM/TSP and 22 individuals not infected with HTLV-1 constituted the healthy subjects group (HS). Pregnant woman and individuals in use of immunosupressing drugs were excluded. The diagnosis of HTLV-1 infection was established by antibody detection by ELISA (Murex HTLV-I+II, Abbot, Dartford, UK) and confirmed by Western blot (HTLV blot 2.4, Genelabs. Singapore). Motor dysfunction and neurological involvement were determined by Osame's motor disability score (OMDS) [37] and Expanded disability status scale (EDSS) [38]. Individuals with an OMDS and EDSS equal to 0 were considered HC. Patients with OMDS ≥1 and presence of specific antibodies against HTLV-1 in the cerebrospinal fluid were diagnosed with HAM/TSP.

### Isolation and culture of peripheral blood mononuclear cells

Peripheral blood mononuclear cells (PBMCs) were obtained from heparinized blood of HTLV-1 infected subjects and healthy controls, and separated by density gradient with Ficoll-Hypaque (GE Healthcare Bio – Sciences, Uppsala, Sweden). PBMCs from the interface were aspirated and washed with saline. After that, these cells were resuspended in RPMI 1640 culture medium with L-glutamine and 25 mM HEPES (Gibco BRL, Grand Island, New York, USA) supplemented with 10% fetal bovine serum (FBS) and 0.5% gentamicin at 10 mg/mL (Gibco BRL, Grand Island, New York, USA). PBMCs were then directed to three separate experiments: a) staining with specific antibodies for analyses of monocyte by flow cytometry; b) culture for determination of spontaneous production of IFN-γ by PBMCs. 3×10^6^ cells/mL were incubated without stimulus or stimulated with PHA (5 µg/mL) at 37°C in 5% CO_2_ for 72 hours and then the supernatant was frozen for later determination of IFN-γ; or c) use for the differentiation of cultured monocytes into macrophages, 5×10^6^ cells/mL. These cells were added to 4-well plates (Lab-Tek Permanox Chamber Slide, Electron Microscopy Sciences, Hatfield, PA) and incubated for 2 hours at 37°C and 5% CO_2_. Cells that did not adhere to the slides were removed by washing. The adherent cells (monocytes) were differentiated into macrophages after 6 days of culture at 37°C in 5% CO_2_, with absence of stimulus, in the presence of LPS or *L. braziliensis*.

### Frequency of monocyte subsets and expression of co-stimulatory molecules

The *ex vivo* frequency of monocyte subsets and expression of HLA-DR, CD80 and CD86 was determined using PBMCs from HC, HAM/TSP patients and HS. Cells were stained with monoclonal antibodies (anti-CD14-FITC, anti-CD16-PE-Cy5, anti-HLA-DR-PE, anti-CD80-PE e anti-CD86-PE, from eBioscience, San Diego, CA or R&D Systems, Minneapolis, MN) for 20 minutes at 4°C. PBMCs were washed with PBS and then fixed with 2% paraformaldehyde. Cells were then analyzed on the flow cytometer (II FacsCanto, BD Biosciences, San Jose, CA). Analysis was performed using FlowJo software version 7.6 (TreeStar, Ashland, OR). The monocyte population was selected based on size and cell granularity and then subdivided into classical (CD14^++^CD16^-^), intermediate (CD14^+^CD16^+^) and non-classical monocytes (CD14^+^CD16^++^).

### Culture and preparation of *Leishmania braziliensis*

A strain of *L. braziliensis* isolated from a patient with cutaneous leishmaniasis from the endemic area of Corte de Pedra, Salvador, Bahia, is maintained, cryopreserved, by the Immunology Service. Parasites were initially cultivated in tubes with biphasic medium (NNN) supplemented with 10% fetal bovine serum and maintained in culture in Schneider medium (LGC Biotechnology, São Paulo, Brazil) supplemented with 10% FBS and 1% penicillin streptomycin and glutamine (Gibco BRL, Grand Island, New York, USA) for expansion and proliferation of protozoa.

### Macrophages infection with *L. braziliensis* and evaluation of macrophages killing

*L. braziliensis* promastigotes were maintained in Schneider medium until reaching stationary (infectious) growth phase. Parasites were than centrifuged and resuspended in RPMI 1640 medium and used to infect macrophages cultured from both HTLV-1 infected subjects and HS. Unstimulated or macrophages stimulated with lipopolysaccharide (LPS) from *Escherichia coli* (100 ng/mL) were used as controls. Infection with *L. braziliensis* was performed at a ratio of 5 parasites to 1 cell for 2 hours at 37°C in 5% CO_2_. Following the incubation period, extracellular parasites were removed by washing, and then the cells were incubated at 37°C and 5% CO_2_. The percentage of macrophages infected with *L. braziliensis* and the number of amastigotes per 100 macrophages were evaluated by optical microscopy after 2, 48 and 72 hours of infection and staining with Giemsa. Counts were performed by two independent observers who were unaware if the slides were from an HTLV-1 infected subject or from a healthy control. The results expressed are the average of the results from both observers.

### Measurement of cytokines and chemokines by ELISA

Culture supernatants from unstimulated PBMCs or from macrophages were collected after 72 and 48 hours of incubation, respectively, and frozen at −20°C until used for determination of cytokines and chemokines. The IFN-γ levels (supernatants of PBMCs) and TNF, IL-10, CXCL9, CXCL10 and CCL5 (macrophages supernatants) were determined by ELISA, using commercial kits and following the manufacturer's instructions (DuoSet R&D Systems, Minneapolis, MN, USA and BD Pharmingen, San Diego, CA, USA). Due to the limited amount of cells some experiments did not included all patients. Data about the production of cytokines and chemokines produced by macrophages after infection by *L. braziliensis* were not represented in the figures, because this pathogen did not induced the production of these molecules.

### DNA extraction and HTLV-1 proviral load

DNA was extracted from 10^6^ cells using proteinase K and salting-out method. The HTLV-1 proviral load was quantified using a real-time TaqMan PCR method [39]. Albumin DNA was used as an endogenous reference. Amplification and data acquisition were carried out using the ABI Prism 7700 Sequence detector system (Applied Biosystems). Standard curves were generated using a 10-fold serial dilution of a double-stranded plasmid (pcHTLV-ALB). All standard dilutions and control and individual samples were run in duplicate for both HTLV-1 and albumin DNA quantification. The normalized value of the HTLV-1 proviral load was calculated as the ratio of (HTLV-1 DNA average copy number/albumin DNA average copy number) ×2×10^6^ and expressed as the number of HTLV-1 copies/10^6^ cells.

### Statistical analyses and data representation

Mann-Whitney test was used to compare IFN-γ production by PBMCs and proviral load between ACs and HAM/TSP patients. Kruskal-Wallis followed by Dunn's post test was used to assess differences between the three groups studied under the same conditions. Wilcoxon test was used to evaluate the influence of stimuli (LPS and *L. braziliensis*) compared to condition without stimulation. Spearman correlation test was used in the correlations results. Data were expressed as median and range (minimum and maximum values). GraphPad Prism 5 (San Diego, CA) was used to carry out the statistical evaluation and a *P*<0.05 was considered to indicate a significant difference.

## Results

Demographic characteristics, IFN-γ production, proviral load and degree of HAM/TSP severity of the participants on this study are shown in Table 1. Of the 45 HTLV-1 infected individuals from an HTLV-1 cohort, 23 were HTLV-1 carriers (HC) and 22 patients had HAM/TSP. There was a predominance of female in both HTLV-1 infected subject's group. Patients with HAM/TSP showed a higher production of IFN-γ by PBMCs than HC (1,979 pg/mL, ranging 38–3,661 pg/mL *vs* 863 pg/mL, ranging 0–3,666 pg/mL, respectively), *P* = 0.03, and also a greater proviral load (231,016 copies/10^6^cells, ranging 932–1.186,254 copies/10^6^cells, *vs* 22,665 copies/10^6^cells, ranging 81–255,319 copies/10^6^cells, respectively), *P* = 0,0004. Patients with HAM/TSP showed a median of OMDS of 6, ranging 3–9 (Table 1).

**Table 1:** Demographic characteristics, IFN-γ production by PBMCs, proviral load and OMDS from HTLV-1 carriers and HAM/TSP patients.

	Healthy subjects (HS) (n = 22)	HTLV-1 carriers (HC) (n = 23)	HAM/TSP patients (n = 22)	*P* value
Age (years)	27 (24–40)	49 (21–71)	57 (35–71)	<0.0001*
Gender (M/F)	9/13 (59%)	5/18 (78%)	2/20 (91%)	0.04**
IFN-γ levels (pg/mL)	0	863 (0–3,666)	1.979 (38–3,661)	0.03***
Proviral load (copies/10^6^cells)	0	22,665 (81–255,319)	231,016 (932–1.186,254)	0.0004***
OMDS			6 (3–9)	

*  =  Kruskal-Wallis; **  =  Chi-square test; ***  =  Mann-Whitney. Data of age, IFN-γ production by PBMCs, proviral load and OMDS are represented by median (minimum and maximum values). Abbreviations: HC: HTLV-1 carriers; HS: Healthy subjects; OMDS: Osame's motor disability score.

### Monocyte subsets frequency and expression of cell surface co-stimulatory molecules

**Figure 1:**
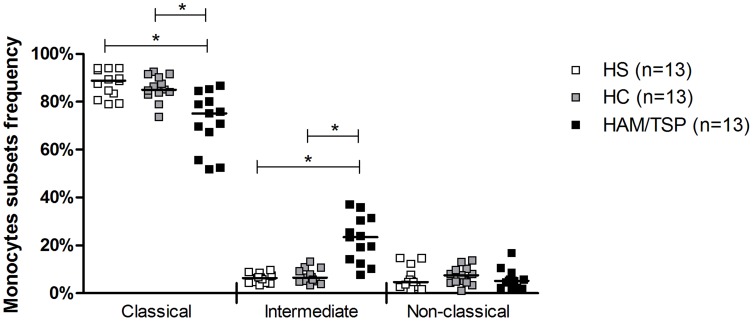
Frequencies of the monocyte subsets. Monocytes from healthy subjects (HS, n = 13), from HTLV-1 carriers (HC, n = 13), and from HAM/TSP patients (n = 13) were selected from the size and cell granularity and then subdivided into classical (CD14^++^CD16^-^), intermediate (CD14^+^CD16^+^) and non-classical monocytes (CD14^+^CD16^++^). Data of the monocyte subsets frequency is represented by median. Kruskal-Wallis followed by Dunn's post test were used for statistical analyses (*P<0.05).

**Figure 2:**
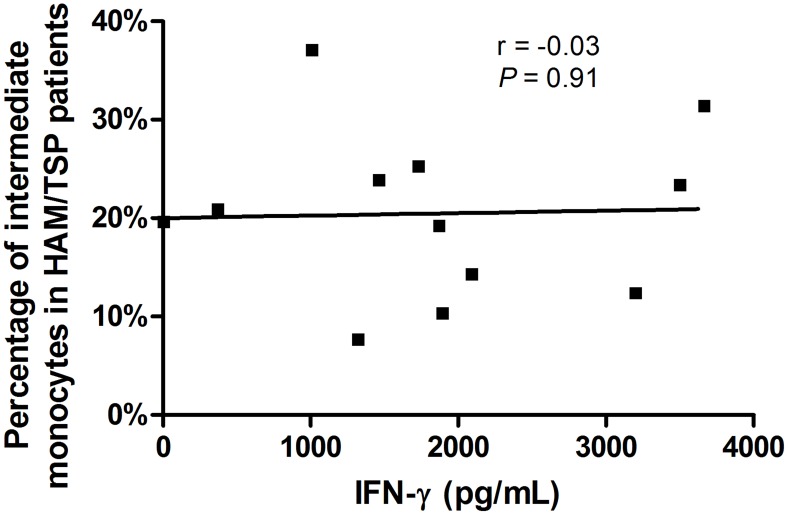
Correlation between IFN-γ produced by PBMCs and intermediate monocytes frequency from HAM/TSP patients. Correlation between IFN-γ production by PBMCs and intermediate monocytes frequency from HAM/TSP patients (n = 12). Spearman correlation and r significance test were used for statistical analyses (*P*<0.05).

The frequencies of monocyte subsets (classical, intermediate and non-classical monocytes) in HC, HAM/TSP patients and HS were determined by flow cytometry and are shown in Fig. 1. The HC group showed a similar frequency of monocyte subsets as observed in the HS group. However, patients with HAM/TSP exhibit lower frequency of classical monocytes and higher frequency of intermediate monocytes than HC and HS. 89.4% of monocytes from HS were classical, while 6.4% were intermediate and 5.1% were non-classical. 83.9% of monocytes from HC were classical, 6.8% were intermediate and 7.5% were non-classical monocytes. HAM/TSP patients showed 75.1% of classical monocytes (*P* = 0.0005 compared to HC and HS), 23.9% of intermediate monocytes (*P*<0.0001 also compared to HC and HS) and 5% of non-classical monocytes (Fig. 1). There was no difference in the expression of HLA-DR, CD80 and CD86 by monocytes between HC, HAM/TSP patients and HS (*P*>0.05). The increasing in frequency of intermediate monocytes in HAM/TSP patients was not associated with IFN-γ production by PBMCs (Fig. 2).

### Macrophage microbicidal ability after *L. braziliensis* infection

**Figure 3:**
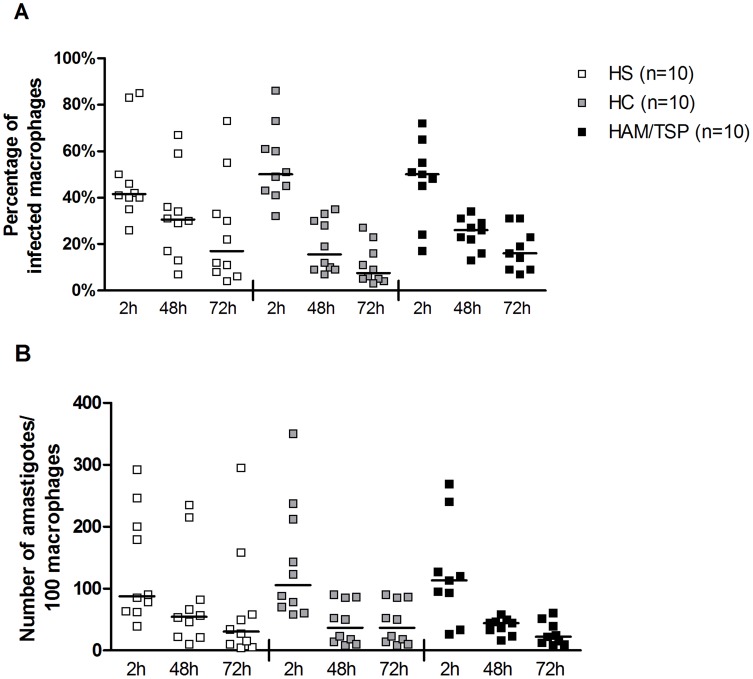
Percentage of infected macrophages and number of amastigotes/100 macrophages from HTLV-1 infected subjects. Macrophages from healthy subjects (HS, n = 10), from HTLV-1 carriers (HC, n = 10) and from HAM/TSP patients (n = 10) were infected by *L. braziliensis* at stationary phase at 5∶1 ratio. (A) Percentage of infected macrophages and (B) the number of amastigotes/100 macrophages were evaluated after 2, 48 and 72 hour of infection. Data is represented by median. Kruskal-Wallis followed by Dunn's post test were used for statistical analyses (*P<0.05).

To evaluate the susceptibility of macrophages from HTLV-1 infected subjects to be infected by an intracellular pathogen and the ability of these cells to kill it, macrophages were infected by *L. braziliensis* at a 5∶1 ratio. The percentage of infected macrophages and the number of amastigotes/100 macrophages were evaluated by optic microscopy after 2, 48 and 72 hours of infection, as shown in Fig. 3. There was no difference in the microbicidal activity between macrophages from the groups studied at any of the three time points following infection by *L. braziliensis*. Macrophages from HTLV-1 infected subjects (both HC and HAM/TSP patients) were initially infected by the parasite at the same proportion as macrophages from HS (Fig. 3A), and there were similar amounts of *L. braziliensis* amastigotes inside the cells after 2, 48 and 72 hours of infection in the groups (Fig. 3B).

### Cytokine and chemokine profile of macrophages in HTLV-1 infection

**Figure 4:**
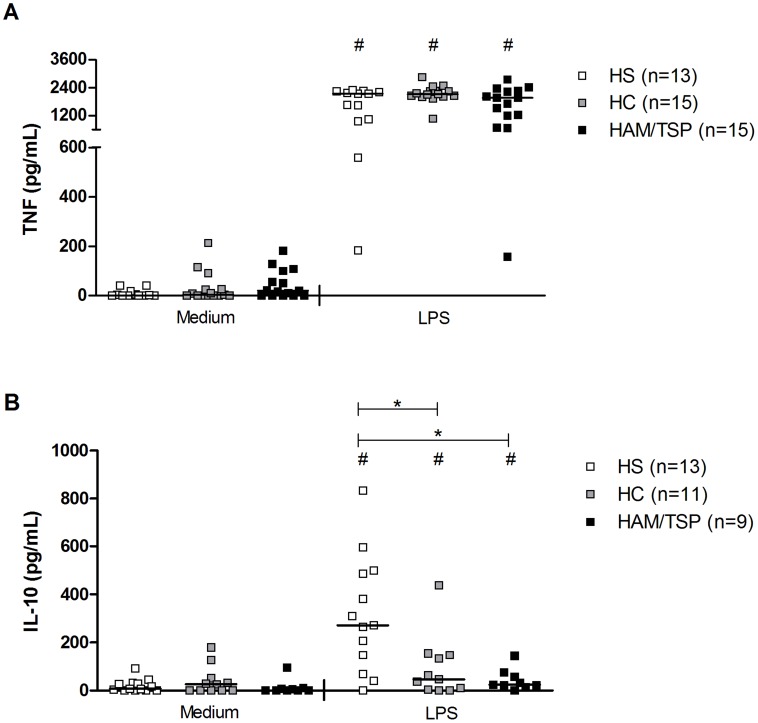
Production of TNF and IL-10 by macrophages from HTLV-1 infected subjects. Macrophages from healthy subjects (HS), HTLV-1 carriers (HC) and from HAM/TSP patients were cultured without stimulus, or with LPS for 48 hours to evaluate (A) TNF and (B) IL-10 productions. ELISA was used to measure these cytokines levels. Data is represented by median and range. Kruskal-Wallis followed by Dunn's post test (* *P*<0.05) and Wilcoxon T test (# *P*<0.05) were used for statistical analyses.

Levels of TNF and IL-10 were evaluated in the supernatants from HTLV-1 infected subjects (HC and HAM/TSP) and HS macrophages cultured after 48 hours of incubation with or without LPS. These assays were performed by ELISA and data are shown in Fig. 4. Neither macrophages from HS, HC nor from HAM/TSP patients produced spontaneously significant detectable levels of TNF (0 pg/mL, 0 pg/mL, 16 pg/mL, respectively). When macrophages from three groups were stimulated with LPS, high levels of TNF were detected (*P*<0.0002). Macrophages from HS produced 2,145 pg/mL while macrophages from HC produced 2,102 pg/mL, and macrophages from HAM/TSP produced 1,965 pg/mL, with no statistically significant differences between those three values (Fig. 4A).

HC, HAM/TSP patients and HS macrophages did not produced IL-10 in significant levels either spontaneously (0 pg/mL, 32 pg/mL and 0 pg/mL, respectively). Upon LPS stimulation, all groups showed increased IL-10 production, but macrophages from HS produced more of this cytokine (265 pg/mL) than macrophages from HC and HAM/TSP patients (46 pg/mL and 31 pg/mL, respectively, *P* = 0.003) (Fig. 4B).

**Figure 5:**
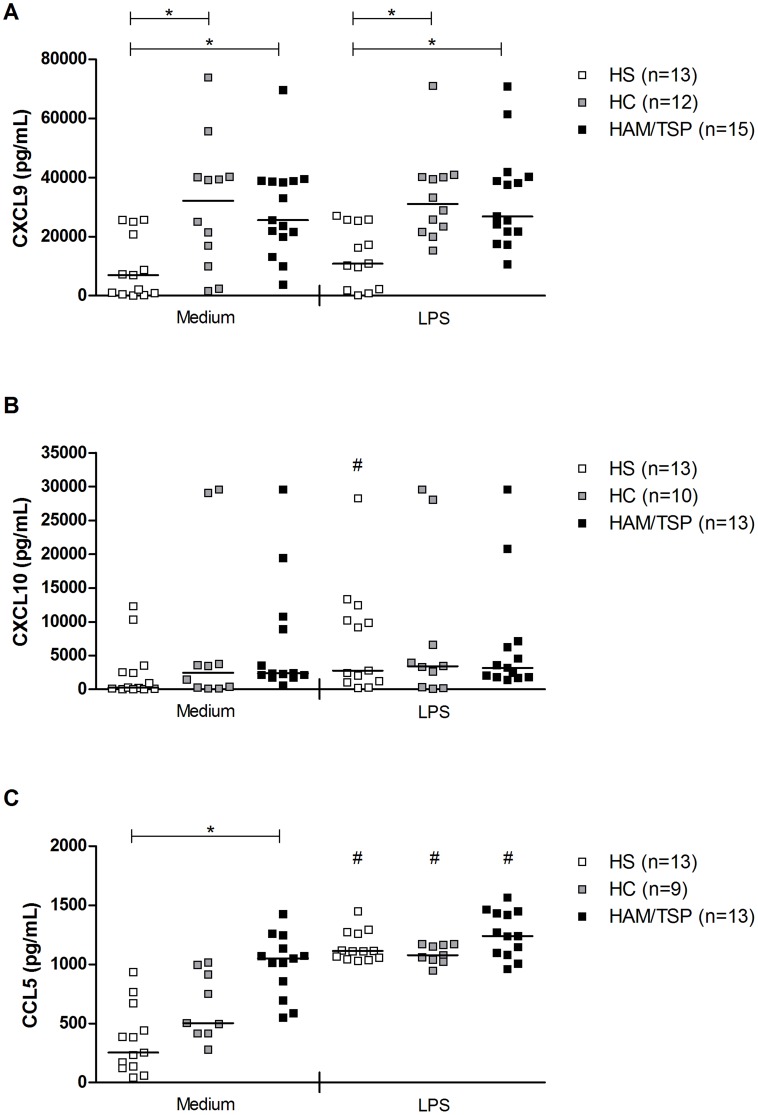
Production of CXCL9, CXCL10 and CCL5 by macrophages from HTLV-1 infected subjects. Macrophages from healthy subjects (HS), HTLV-1 carriers (HC) and from HAM/TSP patients were cultured without stimulus or with LPS for 48 hours to evaluate (A) CXCL9 and (B) CXCL10 and (C) CCL5 productions. ELISA was used to measure these chemokines levels. Data is represented by median and range. Kruskal-Wallis followed by Dunn's post test (* *P*<0.05) and Wilcoxon T test (# *P*<0.05) were used for statistical analyses.

Levels of CXCL9, CXCL10 and CCL5 were evaluated in the supernatants from macrophage cultures of HTLV-1 infected subjects (HC and HAM/TSP patients) and HC after 48 hours of incubation, with or without LPS, as shown in Fig. 5. Macrophages from HC and HAM/TSP patient spontaneously produced more CXCL9 than macrophages from HS (32,115 pg/mL and 25,558 pg/mL *vs* 6,992 pg/mL, *P* = 0.003). Macrophages from HC and HAM/TSP patients also produced more CXCL9 than HS's macrophages after LPS stimulus (31,080 pg/mL and 26,834 pg/mL *vs* 9,648 pg/mL, *P* = 0.001). Furthermore, stimulation with LPS did not induce the production of CXCL9 in macrophages from HS and HTLV-1 infected subjects (Fig. 5A).

Macrophages from HC and HAM/TSP patients produced spontaneously similar levels of CXCL10 (2,458 pg/mL and 2,288 pg/mL) than macrophages from HS (255 pg/mL), After stimulus with LPS, we also did not observed differences between the production of this cytokine by macrophages from HS (2,785 pg/mL), HC (3,418 pg/mL) and HAM/TSP patients (3,201 pg/mL). However, while LPS increased the production of CXCL10 by macrophages from HS compared to the unstimulated condition (*P*<0.03), this stimuli did not increased CXCL10 production by macrophages from HC and HAM/TSP patients (Fig. 5B).

Macrophages from HAM/TSP patients produced more CCL5 than macrophages from HS (1,050 pg/mL *vs* 254 pg/mL, *P*<0.0001). LPS was responsible to increase the production of CCL5 in all group studied (*P*<0.003), but we did not observe statistically significant differences between those groups (Fig. 5C).

### Correlation between IFN-γ produced by PBMCs and proviral load in HTLV-1 infected subjects

**Figure 6:**
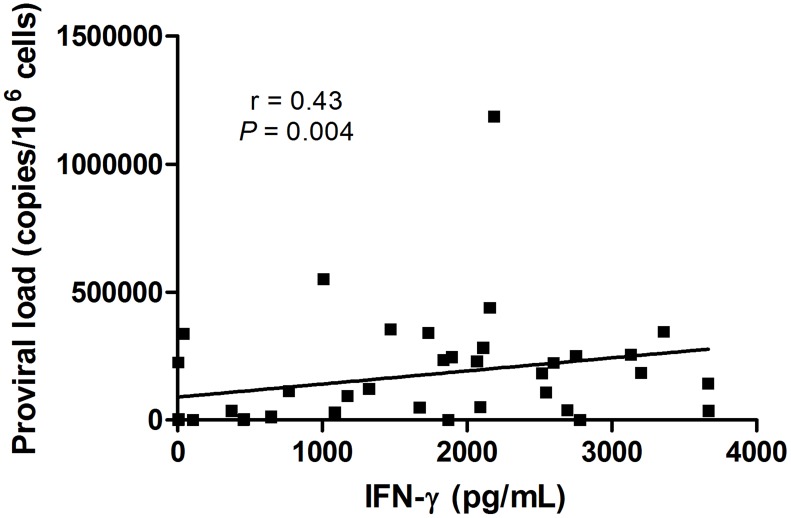
Correlation between proviral load and IFN-γ produced by PBMCs from HTLV-1 infected subjects. Correlation between proviral load of HTLV-1 infected subjects and IFN-γ production by PBMCs (n = 40). Spearman correlation and r significance test were used for statistical analyses (*P*<0.05).

To evaluate if the spontaneous production of IFN-γ by PBMCs was associated with proviral load, correlations were performed using Spearman correlation and r significance test. We observed a direct correlation between IFN-γ and proviral load in HTLV-1 infected subjects, r = 0.43 and *P* = 0.004 (analyzing HC and HAM/TSP patients together) (Fig. 6).

### Correlation between IFN-γ produced by PBMCs and cytokines and chemokines produced by macrophages from HTLV-1 infected individuals

To evaluate if the spontaneous production of IFN-γ by PBMCs was associated with cytokines/chemokines produced by macrophages from HTLV-1 infected subjects, correlations were performed using Spearman correlation and r significance test. We found no correlation between spontaneous production of IFN-γ and production of TNF (r = 0.36 and *P* = 0.08), IL-10 (r = 0.09 and *P* = 0.74), CXCL9 (r = −0.02 and *P* = 0.90), CXCL10 (r = 0.41 and *P* = 0.09) and CCL5 (r = 0.20 and *P* = 0.44) in HTLV-1 infected patients.

### Correlation between proviral load and cytokines/chemokines produced by macrophages from HTLV-1 infected individuals

**Figure 7:**
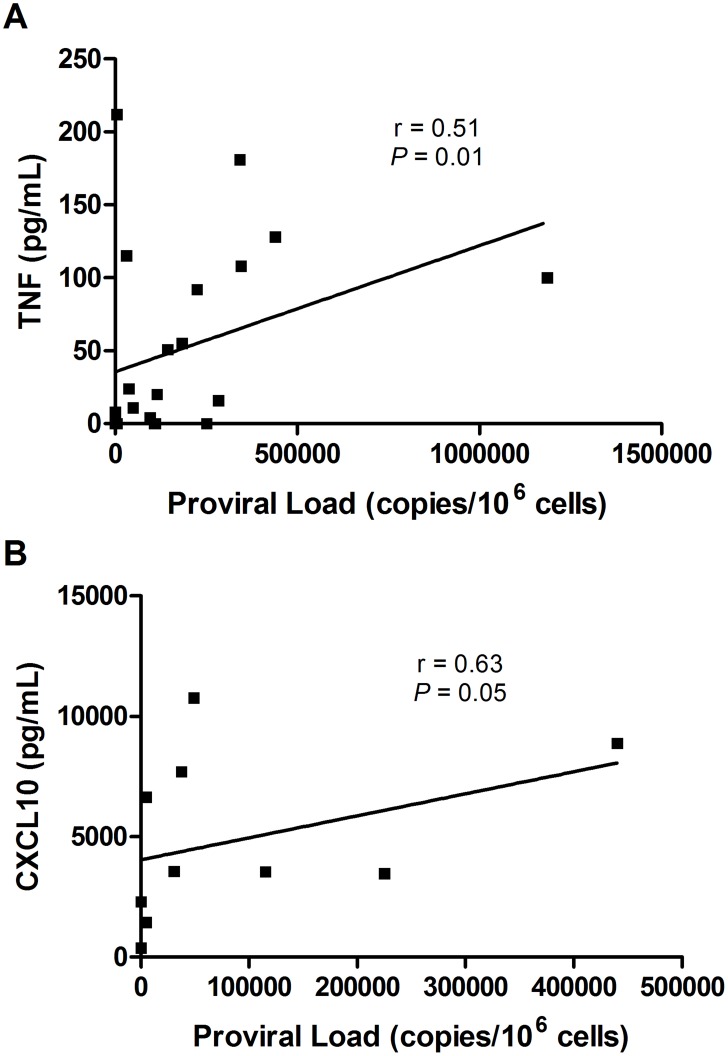
Correlation between proviral load TNF and CXCL10 produced by macrophages from HTLV-1 infected subjects. Correlation between proviral load of HTLV-1 infected subjects and (A) TNF (n = 22) and (B) CXCL10 (n = 10) produced by macrophages. Spearman correlation and r significance test were used for statistical analyses (*P*<0.05).

Correlations between proviral load and cytokines/chemokines produced by macrophages were performed using Spearman correlation and r significance test. We observed a positive correlation, although weak, between proviral load and TNF (r = 0.51 and *P* = 0.01) and CXCL10 (r = 0.63 e *P* = 0.05) (Fig. 7). There was no correlation between proviral load and the IL-10 (r = −0.34 and *P* = 0.26), CXCL9 (r = 0.26 and *P* = 0.24) and CCL5 (r = 0.35 and *P* = 0.21) production by macrophages from HTLV-1 infected individuals.

## Discussion

The activity and phenotype of T cells in HTLV-1 infection have been well studied. These cells are characterized by the increased expression of proinflammatory cytokines, such as TNF and IFN-γ, and increased production of IL-2, which helps maintain CD4^+^ and CD8^+^ T cell proliferation [16], [22]. In contrast very little is known about innate immunity during HTLV-1 infection. We observed that HAM/TSP patients exhibit a higher frequency of intermediate (inflammatory) monocytes than HC and HS, but it was not associated with IFN-γ levels. While the microbicidal ability from HTLV-1 infected subject's macrophages was preserved, macrophages from HTLV-1 infected individuals produced more CXCL9 and CCL5, and less IL-10 than macrophages from HS. Indeed while there was no correlation between IFN-γ and cytokine levels, there was a correlation between proviral loads and TNF and CXCL10 production.

It is known that monocytes are a heterogeneous population of cells and can be classified, based on the expression of CD14 and CD16, as classical, intermediate or inflammatory and non-classical. Here we documented that HAM/TSP patients have a higher frequency of intermediate monocytes than HC and HS. It is known that intermediate monocytes are the main source of TNF among the three subpopulations [40]. As PBMCs from HTLV-1 infected subjects, especially from HAM/TSP patients, produce more proinflammatory mediators such as CXCL9, CXCL10 and TNF than PBMCs from HS [16], [41], we hypothesized that monocytes may play an important role in the inflammatory response and in the pathogenesis of HAM/TSP. Agreeing with this hypothesis, we observed that there was no correlation between IFN-γ production and increasing intermediate monocytes.

Macrophages are capable of killing infectious agents but may also serve as habitat for intracellular pathogens. As HTLV-1 infection increases the susceptibility to infections caused by intracellular agents such as *M. tuberculosis*, we sought to evaluate macrophage microbicidal function in HTLV-1 infection. To evaluate macrophage killing we used *L. braziliensis*, an intracellular pathogen knowing to interact with TLR2, TLR4 and TLR9 [42], [43] and with the ability to multiply in macrophages. As the number of intracellular parasites inside the macrophages after 2 hours of infection was similar to that observed in HS, it was concluded that penetration and/or phagocytosis of *L. braziliensis* was equal in the three groups. Moreover the leishmania killing, evaluated at 48 hours and 72 hours by quantifying the number of intracellular amastigotes in macrophages, was similar. This data extend our previous observations that the ability of neutrophils from HTLV-1 infected subjects to kill leishmania parasites is preserved [32]. As IFN-γ is the main cytokine known to activate macrophages and high IFN-γ production is observed in HTLV-1 infected subjects, one could expect that macrophages from HAM/TSP patients had greater ability to kill an intracellular pathogen, but we did not find that the marked Th1 environment observed in these individuals modified the killing ability of myeloid cells.

Macrophages activation has been used to indicate both, increasing ability of killing and secretion of molecules such as chemokines and cytokines. Macrophages are also a heterogeneous cells population and macrophage's subsets have been defined as classical macrophages that are associated with a type 1 immune response, and alternative macrophages that secret IL-4 and IL-10 [44]. Here we showed that the killing ability and secretion of cytokines by macrophages are not necessarily associated. While we did not observe an increase in the killing ability of macrophages from HTLV-1 infected subjects, manly in unstimulated cells or after LPS stimulation, macrophages produced higher levels of CXCL9 and CCL5 than HS's macrophages. This indicates that at the level of innate immunity, there was an enhancement of chemokines related with both Th1 and Th2 immune responses. This is an agreement with the observation that atopic diseases may occur in HTLV-1 infection [30] and that PBMC from HC produce higher amount of Th2 cytokines than cells from HS [16]. In this study we did not observed a higher production of CXCL10 by macrophages from HC and HAM/TSP patients compared to HS, but while the cells from HS were stimulated by LPS to produce this chemokine, it was not observed in macrophages from HC and HAM/TSP patients. A reasonable explanation for this observation is that cells from HTLV-1 infected subjects could have already reached the limit of production of these chemokine even before the addition of LPS in the cultures.

It's known the ability of LPS to induce strong TNF production which is followed by IL-10 synthesis. The observation that HS's macrophages stimulated with LPS produced more IL-10 than cells from HTLV-1 infected subjects, both HC and HAM/TSP patients, suggests an impairment on macrophages of these individuals to secret this regulatory cytokine. Although the high production of proinflammatory mediators documented in HTLV-1 infection, especially in HAM/TSP patients, such as IFN-γ, IL-1, IL-6, could contribute to the increased production of chemokines and TNF by macrophages, we did not find a correlation between the IFN-γ production by PBMCs and TNF, IL-10, CXCL9, CXCL10 and CCL5 produced by macrophages. In contrast with the absence of correlation between IFN-γ and cytokines/chemokines levels, there was a direct correlation between proviral load and TNF and CXCL10 levels. This indicates that the HTLV-1 by itself or by inducing soluble mediators play a key role in the increased ability of macrophages to produce cytokine during HTLV-1 infection. Therefore it is important that future studies evaluate not only the role of viral proteins and other viral factors in activate innate immunity cells but also in disease expression associated to HTLV-1.

It is already known that PBMCs from HAM/TSP patients produced more IFN-γ and have a higher proviral load compared to HC [16], [17], [45]. In agreement with previous observations, we also documented a positive correlation between proviral load and IFN-γ production by PBMCs from HTLV-1 infected subjects. However our data clearly show while the IFN-γ production did not correlate with the increased cytokine/chemokine production by macrophages in HTLV-1 infected subjects, there was a correlation between proviral load and TNF and CXCL10.

This is the first study to evaluate the monocyte subsets and activation, as well as the inflammatory and microbicidal activity from macrophages in HTLV-1 infection. Our data indicate that patients with HAM/TSP have an increase frequency of intermediate monocytes. We also observed that macrophages from HTLV-1 infected subjects have the same ability to phagocytize and kill an intracellular pathogen as healthy subject's macrophages, but that proinflammatory activity was enhanced in HTLV-1 infected subjects. The dissociation between microbicidal activity and production of proinflammatory cytokines and chemokines is a relevant subject and show that inflammation and killing may be independent functions. As IFN-γ is the main cytokine that activate macrophages we expected at some extension a relationship between this cytokine and the inflammatory profile observed in monocytes and macrophages. However the absence of correlation between IFN-γ and cytokine/chemokine production and a direct correlation between proviral load and TNF and CXCL10 produced by macrophages suggests innate immune cells triggered by viral factors may play an important role in the inflammatory response and in the pathogenesis of HAM/TSP.
